# Seasonal Regulation of Metabolism: The Effect of Wintertime Fasting and Autumnal Fattening on Key Central Regulators of Metabolism and the Metabolic Profile of the Raccoon Dog (*Nyctereutes Procyonoides*)

**DOI:** 10.3390/ijms22094965

**Published:** 2021-05-07

**Authors:** Laura Niiranen, Kari A. Mäkelä, Anthony Dona, Jan Krumsiek, Toni Karhu, Markus J. Mäkinen, Olaf Thalmann, Seppo Saarela, Karl-Heinz Herzig

**Affiliations:** 1 Research Unit of Biomedicine, Faculty of Medicine, University of Oulu, P.O. Box 5000, FIN-90014 Oulu, Finland; laura.niiranen@oulu.fi (L.N.); kari.makela@oulu.fi (K.A.M.); karhu.toni@gmail.com (T.K.); othalmann@ump.edu.pl (O.T.); 2 Kolling Institute of Medical Research, University of Sydney, St Leonards, NSW 2065, Australia; anthony.dona@sydney.edu.au; 3 Department of Physiology and Biophysics, Institute for Computational Biomedicine, Englander Institute for Precision Medicine, Weill Cornell Medicine, New York, NY 10065, USA; jak2043@med.cornell.edu; 4 Cancer and Translational Medicine Research Unit, Department of Pathology, University of Oulu, P.O. Box 5000, FIN-90014 Oulu, Finland; markus.makinen@oulu.fi; 5 Medical Research Center Oulu, P.O Box 8000, FIN-90014 Oulu, Finland; 6 Department of Pathology, Oulu University Hospital, P.O. Box 5000, FIN-90014 Oulu, Finland; 7 Institute of Pediatrics, Poznań University of Medical Sciences, 60-572 Poznań, Poland; 8 Ecology and Genetics Research Unit, Faculty of Science, University of Oulu, P.O. Box 3000, FIN-90014 Oulu, Finland; seppoyo.saarela@gmail.com

**Keywords:** winter sleep, orexigenic neuropeptides, anorexigenic neuropeptides, food intake, metabolome, fasting

## Abstract

Investigations into the mechanisms regulating obesity are frantic and novel translational approaches are needed. The raccoon dog (*Nyctereutes procyonoides*) is a canid species representing a promising model to study metabolic regulation in a species undergoing cycles of seasonal obesity and fasting. To understand the molecular mechanisms of metabolic regulation in seasonal adaptation, we analyzed key central nervous system and peripheral signals regulating food intake and metabolism from raccoon dogs after autumnal fattening and winter fasting. Expressions of neuropeptide Y (NPY), orexin-2 receptor (OX2R), pro-opiomelanocortin (POMC) and leptin receptor (ObRb) were analyzed as examples of orexigenic and anorexigenic signals using qRT-PCR from raccoon dog hypothalamus samples. Plasma metabolic profiles were measured with ^1^H NMR-spectroscopy and LC-MS. Circulating hormones and cytokines were determined with canine specific antibody assays. Surprisingly, NPY and POMC were not affected by the winter fasting nor autumn fattening and the metabolic profiles showed a remarkable equilibrium, indicating conserved homeostasis. However, OX2R and ObRb expression changes suggested seasonal regulation. Circulating cytokine levels were not increased, demonstrating that the autumn fattening did not induce subacute inflammation. Thus, the raccoon dog developed seasonal regulatory mechanisms to accommodate the autumnal fattening and prolonged fasting making the species unique in coping with the extreme environmental challenges.

## 1. Introduction

A lack of food sources is a common challenge for wild animals and surviving in such conditions requires resilience to fasting periods, saving energy by the hypometabolism and storing energy as food caches or as adipose tissue in the body. Today mankind is facing the opposite problem as high energy food sources are easily available, lifestyles have changed to be less strenuous and the physical activity intensity is reduced. The result of high input via food and low output by less physical activity culminates in the increasing prevalence of obesity worldwide with its significant health risks, such as insulin resistance, dyslipidemia, high blood pressure, cancer and dementia. In obesity, adipose tissue is accumulated in an excessive manner, causing these adverse health effects [1,2,3,4,5]. Intriguingly, in nature some animal species repeatedly undergo non-pathological large-scale fluctuations in their body fat storages and energy metabolism as a part of their natural seasonal cycles without major adverse effects to their health. It is therefore prudent to investigate the molecular mechanisms regulating these opposite metabolic states.

The raccoon dog (*Nyctereutes procyonoides*) is a Eurasian middle-sized canid species, which even thrives in various challenging environmental conditions [6,7]. It is omnivorous and has thick fur that offers protection from the cold weather. During winter time the raccoon dog undergoes a relatively shallow form of hibernation with only a slight body temperature reduction of 0.5–1.5 °C [8,9,10] as compared to other animal species that can undergo a more complete metabolic depression. Raccoon dogs maintain a certain level of activity throughout winter rest and the winter lethargy can be discontinued by longer periods of alertness when the animals can venture out of the dens to defecate and even forage for food if the snow cover allows it [8,11]. The winter season is often preceded by a period of autumnal fattening, during which the animals feed mostly on berries, grains and small mammals, gaining up to 30–40% weight in white adipose tissue (WAT) [12]. The abundant WAT deposits enable the raccoon dogs to maintain long fasting periods of up to 11 weeks [13], while they effectively maintain their bone and muscle mass through unknown mechanisms [14,15].

Food intake and body weight are mainly regulated by the hypothalamus that acts as a homeostatic switch for various autonomic functions such as thermoregulation, reproduction, hormone secretion, sleep and circadian rhythms [16,17,18]. Food intake is regulated by the activation and inhibition of special neurons located in the hypothalamic nuclei with their orexigenic and anorexigenic peptides. The arcuate nucleus of the hypothalamus contains the orexigenic NPY and agouti-related protein (Agrp), and the anorexigenic POMC and cocaine and amphetamine-regulated transcript (CART) expressing neurons [19,20]. Another important hypothalamic area participating in the control of food intake is the lateral hypothalamus, containing orexigenic neurons. Orexins are considered potential regulators of the winter lethargy and body weight cycles since they are involved in the regulation of wakefulness and arousal [21,22,23,24]. Thus, orexins are intriguing candidates for the regulating mechanism for food intake in an animal species with seasonally regulated physiology. Circulating leptin secreted by WAT is an important satiety signal to the central nervous system (CNS), indicating a positive energy balance and accumulation of energy storages [25,26]. Leptin is transported via blood to CNS where it passes through the blood brain barrier to the hypothalamus by saturated transport mechanisms, inhibiting food intake. Also, the interplay of the gut-brain axis plays an important role in regulating satiety, food intake and metabolism. The hypothalamus receives signals of digesting and processing food from the gastrointestinal tract (e.g., peptide YY, ghrelin, cholecystokinin, glucose-dependent insulinotropic peptide) [27,28,29], which are likely modulated during the seasonal hyperphagia and fasting in the raccoon dog. In addition, signals from processed food directly affect food intake and the fluctuations in energy levels can be captured at the metabolic level. The metabolome includes all the small-molecule metabolites resulting from the ongoing cellular processes [30] and thus reflects the actively used metabolic pathways and the whole organism energy status at a certain time point.

It has been suggested that the satiety promoting signals are down-regulated after the autumnal fattening to accommodate the winter hibernation and up-regulated as the animals emerge from the hibernation to initiate food intake [31,32,33]. The obligate hibernating thirteen-lined ground squirrel (*Ictidomys tridecemlineatus*) undergoes daily torpor bouts during the hibernation period and begins to down-regulate the hypothalamic orexigenic and energy storing stimulating peptides such as NPY after the autumnal hyperphagic phase [34,35,36]. Orexigenic hypothalamic peptides NPY, Agrp and orexins are upregulated after the hibernation period to initiate food intake [36]. Thus, our hypothesis is that food intake regulating hypothalamic peptides follow a pattern resembling non-hibernating euthermic species in which the hypothalamic orexigenic peptides are upregulated during fasting conditions and downregulated when there is a positive energy balance, whereas the anorexigenic signals would be affected in an opposite manner.

In order to test the hypothesis, we investigated the role of key central nervous system food intake regulators and the impact on the metabolome during autumnal fattening and prolonged winter fasting in the raccoon dog.

## 2. Results

### 2.1. Body Mass

The winter fasted and winter fed group raccoon dogs weighed 13.3 ± 1.1 kg and 14.0 ± 1.2 kg, respectively, at the beginning of the experiment. At the end of the experiment, the body mass of the winter fasted was 9.0 ± 0.9 kg, winter fed 11.2 ± 0.9 kg and autumn fed 12.3 ± 1.2 kg. Winter fasted raccoon dogs lost 32.2% and winter fed 20% of their body weight during the experiment. The winter fasted weighted significantly less than the winter fed (*p* = 0.009 **) and autumn fed (*p* = 0.000 ***) animals at the end of the experiment (Table 1). The prolonged food deprivation during the 10 weeks of fasting was well tolerated without other adverse health effects. There was no significant difference in the body mass between the winter fed and autumn fed raccoon dogs.

### 2.2. Hypothalamic Peptides and Receptors

**Figure 1:**
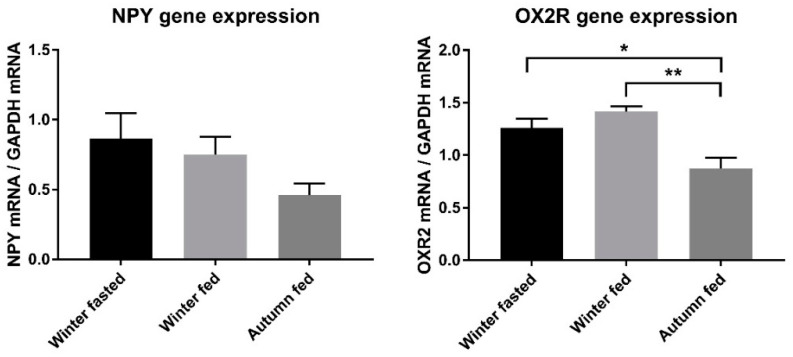
Relative mRNA level expression of orexigenic NPY and OX2R normalized to the expression of GAPDH in raccoon dog hypothalamus. Statistically significant differences are indicated with * *p* < 0.05, and ** *p* ≤ 0.01.

**Figure 2:**
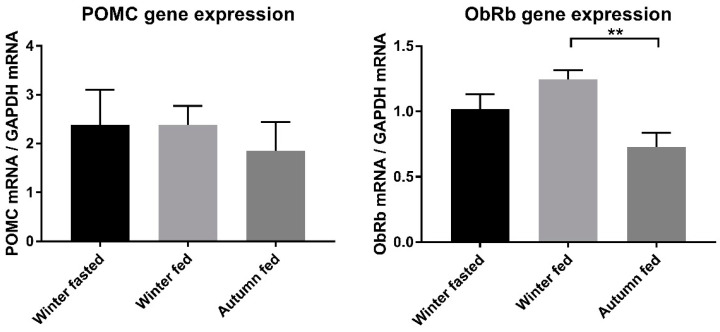
Relative mRNA level expression of anorexigenic POMC and ObRb normalized to the expression of GAPDH in raccoon dog hypothalamus. Statistically significant difference is indicated with ** *p* ≤ 0.01.

Expression of OX2R in the hypothalamus was significantly reduced in autumn fed animals compared with winter fed by 38.2% (*p* = 0.002 **) and in winter fasted by 30.7% (*p* = 0.029 *) compared with winter fed (Figure 1). The autumn fed had also a significantly smaller relative expression of ObRb in the hypothalamus compared to winter fed by 35.1% (*p* = 0.005 **) (Figure 2). To our surprise, the relative expression of hypothalamic NPY and POMC did not significantly differ between the groups.

### 2.3. Plasma Hormones and Inflammatory Markers

The ten-week winter fasting period did not significantly affect plasma metabolites and cytokine/chemokine profiles as compared with the winter fed group. Furthermore, in the autumn fed group, plasma metabolites and chemokines/cytokines were similar to in the winter fasted and winter fed groups (Table 2). Glucagon-like peptide 1 (GLP-1), brain-derived neurotrophic factor (BDNF), thyroid-stimulating hormone (TSH), interferon gamma (IFN-γ), interleukin 4 (IL-4), interleukin 10 (IL-10), interleukin 6 (IL-6) and tumor necrosis factor (TNFα) were under assay detection limits. Minimum detectable concentrations of the assays were 7.7 pg/mL for GLP-1, 8.19 pg/mL for BDNF, 0.79 ng/mL for TSH, 4.4 pg/mL for IFN-γ, 28.8 pg/mL for IL-4, 1.6 pg/mL for IL-10, 12.1 pg/mL for IL-6 and 0.4 pg/mL for TNFα.

#### Histopathology of Adipose Tissue Vascularization and Inflammatory Structures

**Figure 3:**
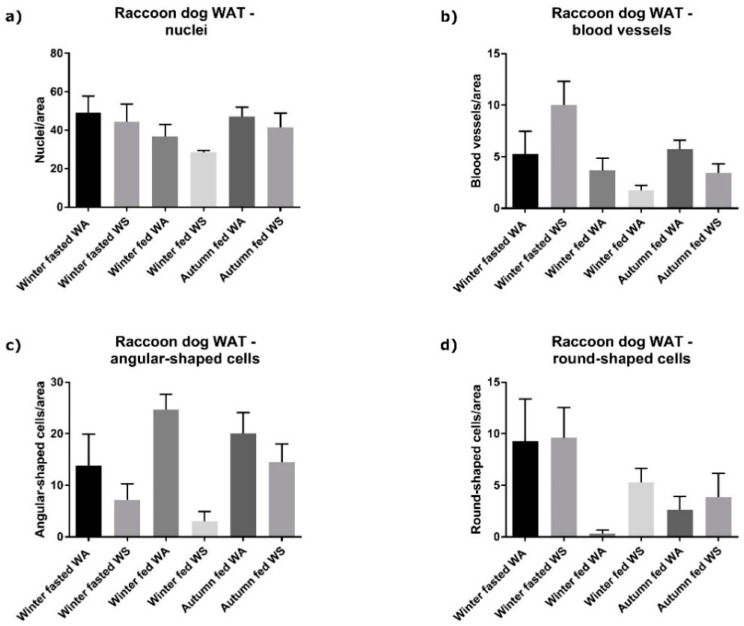
Histopathological analysis of raccoon dog WAT samples. (**a**) Amount of nuclei per area, (**b**) vascularization as amount of blood vessels per area and (**c**,**d**) shape profile of the adipocytes.

The amount of white adipose tissue nuclei did not significantly change between the raccoon dog groups or abdominal (WA) and subcutaneous (WS) deposit locations, indicating that there was no adipocyte hyperplasia (Figure 3a). The size measurement of adipocytes could unfortunately not be done from the frozen samples. Vascularization of WS showed a higher trend in the winter fasted raccoon dogs, but this was not statistically significant (Figure 3b). There were also no significant differences in the adipocyte shape profiles, although WAT from the abdominal cavity seems to be more abundant with the angular-shaped adipocytes than the subcutaneous WAT (Figure 3c,d). Angular cells were more prominent in the WA and WS in the autumn fed and in WA in the winter fed, compared to the winter fasted. Similarly, the round shape was more frequently seen in the winter fasted animals and in general in subcutaneous fat. The shape of the adipocytes suggests a more tightly organized pattern with angular-shaped cells, whereas the round shape indicates a more loose structure. No inflammatory cells or crown-like structures were detected in any of the adipose tissue histopathological samples.

### 2.4. ^1^H NMR Analysis

**Figure 4:**
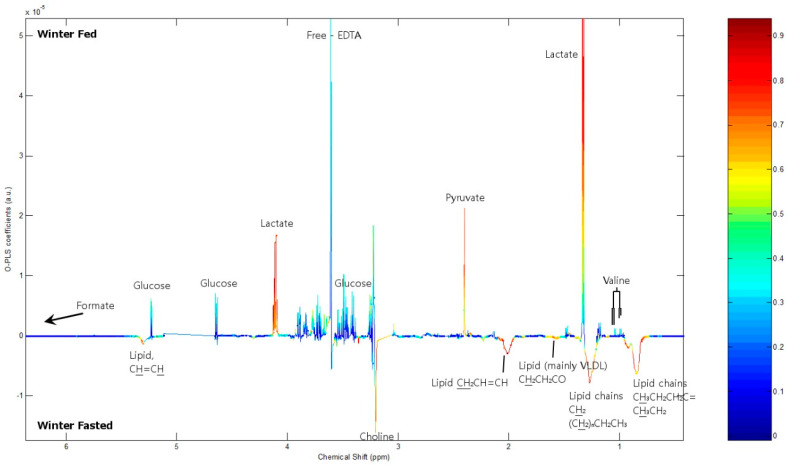
Loadings obtained by an OPLS-DA multivariate analysis on the NMR spectra comparing winter fed to winter fasted. The height of the peak represents the abundance of the metabolite in either group and the warmer coloring scheme (yellow, orange, red) indicate higher correlation value up to 1 within the group. The metabolite assignments are added in the Figure for visualization. Hyphenations in the Figure are EDTA, ethylenediaminetetraacetic acid and VLDL, very low-density lipoprotein.

**Figure 5:**
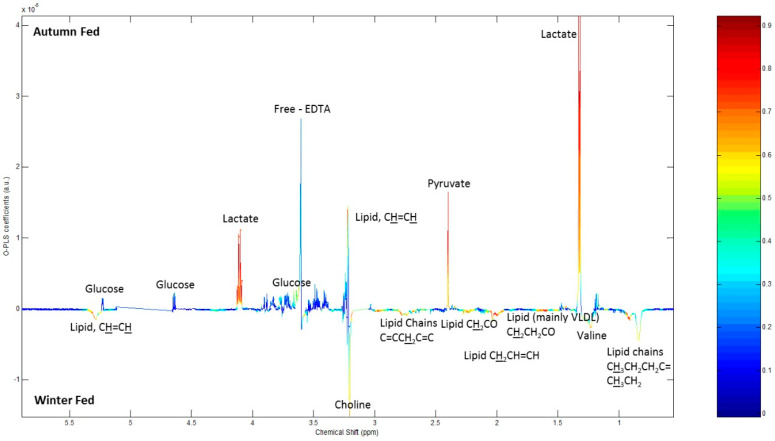
Loadings obtained by an OPLS-DA multivariate analysis on the NMR spectra comparing autumn fed to winter fed. See Figure 4 caption for interpretation.

**Figure 6:**
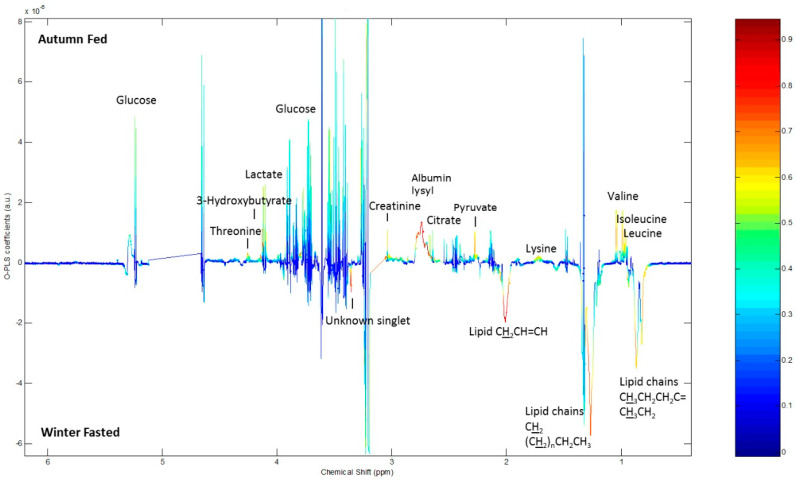
Loadings obtained by an OPLS-DA multivariate analysis on the NMR spectra comparing autumn fed to winter fasted. See Figure 4 caption for interpretation.

According to the ^1^H NMR spectroscopy analysis of the raccoon dog plasma, the plasma of winter fed contained higher concentrations of lactate and pyruvate with a good correlation within the group compared with winter fasted. Winter fasted group plasma had more unsaturated lipoprotein, saturated mobile lipids and choline compared to winter fed (Figure 4). Autumn fed raccoon dog plasma exhibited more lactate and pyruvate with a good correlation compared to winter fed, whereas the winter fed groups plasma contained more choline, unsaturated lipoproteins and saturated lipids (Figure 5). When comparing autumn fed raccoon dog plasma to winter fasted plasma, the spectrum profiles showed more variability, but mostly with a low correlation within the groups. The winter fasted exhibited more unsaturated lipoproteins compared with the autumn fed whereas the autumn fed had more albumin lysyl (Figure 6). Lactate, pyruvate, glucose and branched-chain aminoacids (BCAA) valine, isoleucine and leucine were more prominent in the autumn fed compared with the winter fasted, but with a weaker correlation coefficient. The greatest differences between the groups arose from unsaturated lipoproteins.

### 2.5. UPLC-MS Analysis

**Figure 7:**
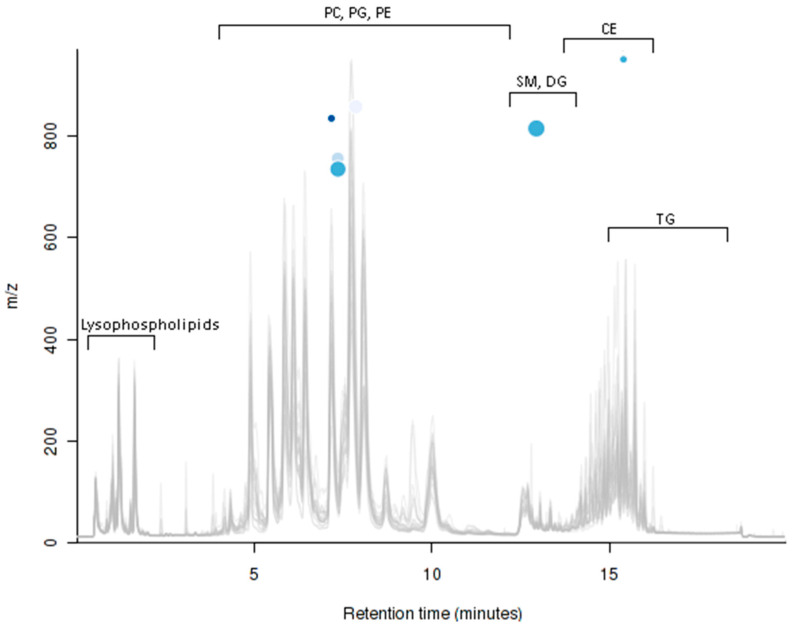
Cloudplot of the multigroup comparisons of the total ion chromatograms of winter fasted, winter fed and autumn fed with metabolic data visualization in the positive ionization mode. The six blue spheres represent the variably expressed features between the groups. The color of the sphere indicates the *p*-value of the feature (the lower the *p*-value the darker the color) and the larger diameter indicates the fold change, including the abundance and intensity of the feature (the larger the diameter, the larger intensity of the feature). Hyphenations in the Figure: PC, phosphatidylcholines; PG, phosphatidylglycerols; PE, phosphatidylethaloamines; SM, sphingomyelins DG, diacylglycerols; CE, cholesterol esters and TG, triacylglycerols are denoting the common retention times in the system for the different metabolites, which were added in the Figure for visualization.

In the positive ionization mode UPLC-MS analyses of a total of 510 aligned ion features, multigroup comparisons resulted in six significantly differentially expressed features (*p* ≤ 0.05) between at least two of the compared groups. Three of the significantly different ion features were isotopes and three were annotated as actual host/parent molecules. The six variably expressed features are presented in a cloudplot with the metabolomics data visualization package (Figure 7) and the three variably expressed parent molecules are presented in box-and-whiskers plots along with the post hoc comparisons and putative database identifications based on accurate m/z in the supplementary attachments. Metabolite in the sphingolipids class was significantly higher in winter fed compared with the autumn fed raccoon dogs (Figure S1: Box- and whisker-plot 1). In addition, a metabolite in the glycerophospholipids class was significantly higher in the winter fasted compared with autumn fed (Figure S2: Box- and whisker-plot 2) and in the winter fed compared with the winter fasted (Figure S3: Box- and whisker-plot 3). The ^1^H NMR spectroscopy analysis results suggest that choline residues (glycerophosphocholine, diacylglycerophosphocholine (Excel S1)) were more abundant in the fasted raccoon dogs than in the fed groups (Figure 4, Figure 5 and Figure 6).

**Figure 8:**
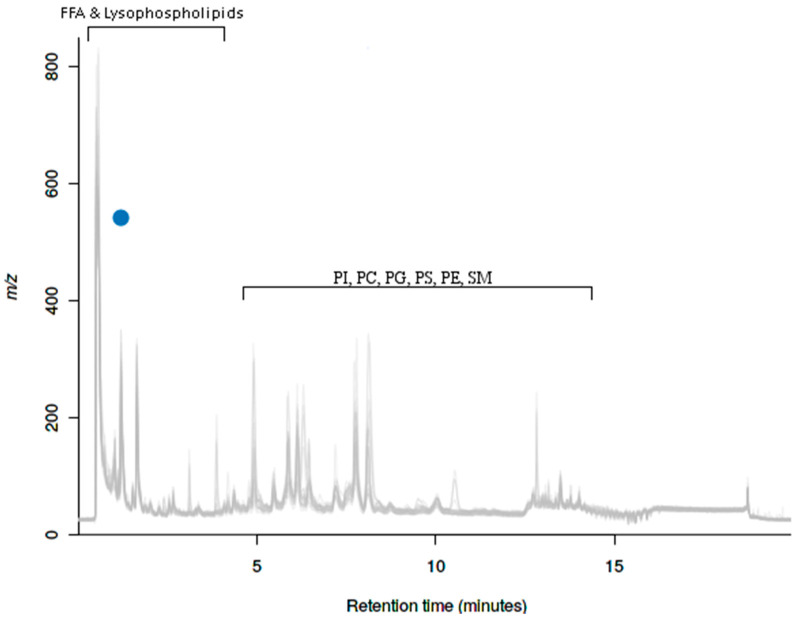
Cloudplot of the multigroup comparisons of the total ion chromatograms of winter fasted, winter fed and autumn fed with metabolic data visualization in the negative ionization mode. The two blue spheres represent the variably expressed features between the groups. The color of the sphere indicates the p-value of the feature (the lower the p-value the darker the color) and the larger diameter indicates the fold change e.g. the abundance and intensity of the feature (the larger the diameter the larger intensity of the feature). Hyphenations in the Figure: FFA, free fatty acids; PI, phosphatidylinositols; PS, phosphatidylserines; are denoting the common retention times in the system for the different metabolites, which were added in the Figure for visualization.

The datasets of the winter fasted, winter fed and autumn fed group raccoon dogs contained in total 78 negatively charged ion features. In multigroup comparisons, two features were found to be significantly (*p* ≤ 0.05) differentially expressed between at least two of the compared groups. One of the two differentially expressed features was an isotope and the second was a parent molecule. The two variably expressed features are presented in a cloudplot (Figure 8). The differentially expressed parent molecule is presented in a box-and-whiskers plot with post hoc comparisons between the compared groups and putative database annotations. The feature with m/z 541.3 was identified as penta amino acid with the databases and it was more abundant in the two winter groups (winter fasted and winter fed) compared with the autumn fed (Figure S4: Box- and whisker-plot 4). A more extensive list on metabolite identification on the basis of m/z from several metabolite databases can be found in the Supplementary Information (Excel S1).

## 3. Discussion

In this study, we determined central and peripheral signals that regulate food intake and metabolism in raccoon dogs under seasonal fattening and fasting conditions. The two winter groups exhibited differences in hypothalamic OX2R and ObRb compared with the autumn group, indicating seasonal regulation, whereas the effects of the prolonged ten-week fasting period showed a weaker response despite the significantly lower body weight of the animals. Orexigenic OX2R expression was significantly higher in winter fed and fasted groups compared with the autumn fed group, which could participate in the promotion of wakefulness and food intake. The higher OX2R expression in the two winter groups, sampled in the beginning of March with already significant lengthening of daylight as compared to its shortening in the autumn group, may reflect a seasonal transition to initiate food intake after winter. Similar changes have been reported in the hibernating thirteen-lined ground squirrel (*Ictidomus tricemlineatus*) which exhibited low levels of the orexigenic NPY, orexins and Agrp in the hypothalamus during hibernation, but started to increase the levels in early spring after emerging from hibernation to restart food intake behavior [36]. Our results show no statistical difference in the relative hypothalamic expression of the orexigenic NPY between the different raccoon dog groups, suggesting that the NPY levels were maintained at quite stable level despite the prolonged ten-week fasting period and the autumnal fattening phase. A previous study on Siberian hamsters (*Phodopus sungorus*) showed that 48 h of fasting increased hypothalamic NPY concentrations similar to those of the non-seasonal mammals [34], but induced no changes in hypothalamic orexin or POMC after the fasting both in long and short day length [35]. Mercer et al. [37] found that 18 weeks into short day exposure, Siberian hamster decreased the hypothalamic POMC and increased Agrp, but no effect on NPY could be detected. A further superimposed 24 h fasting increased NPY and Agrp expression without any further effect on POMC expression.

The raccoon dogs in the autumn fed group exhibited significantly less hypothalamic ObRb in comparison to the winter fed group, suggesting a seasonal effect in the regulation. The downregulation of leptin receptors at the end of the autumnal fattening period might be an adaptation to maintain and enlarge the leptin secreting WAT storages. In accordance with the significantly larger body weight, circulating plasma leptin levels were significantly higher in the autumn fed raccoon dogs compared to the winter fed and winter fasted [38]. In addition, the reduced expression of the ObRb in the hypothalamus could contribute to the energy intake to induce autumn fattening before the winter [39]. Leptin inhibits the orexigenic NPY/Agrp expressing neurons and stimulates the anorexigenic POMC/CART neurons in the arcuate nucleus of the hypothalamus in a dual action to reduce food intake [40]. These neuron systems have projections to second order neurons in the medial and lateral hypothalamus, which include orexin producing neurons. Thus, the high circulating leptin levels in the autumn fed raccoon dogs could contribute to the lower OX2R expression in the autumn fed group as compared to the winter fed and winter fasted and to the lower hypothalamic NPY expression in the autumn fed compared to the winter fasted dogs.

In the raccoon dogs, food deprivation did not significantly affect hypothalamic orexigenic signals NPY and OX2R as seen in rodents, several hibernating and euthermic species and humans [41,42,43]. In addition, anorexigenic POMC or ObRb expression were not changed by the prolonged fasting period, indicating that hunger feeling might be suppressed during food scarcity. The seasonal fluctuation in adipose tissue storages enables them to survive long fasting periods without altering the hypothalamic orexigenic and anorexigenic peptide expressions. The lower expression of hypothalamic ObRb in autumn fed raccoon dogs compared with the winter fed group and the lower hypothalamic OX2R expression compared with both winter fasted and fed groups suggest that the seasonal effects were more potent than the availability of food during the winter.

The levels of plasma hormones suggest that the raccoon dogs are well adapted to the ten-week fasting period as well as to the autumnal fattening. The pituitary derived peptides (ACTH, GH, FSH, LH, TSH), adipose tissue derived resistin, and gut peptides GIP and PYY showed no differences between the groups. Plasma cytokine and chemokine levels of GM-CSF, IP-10, IL-6, IL-8, IL-18 and MCP-1 remained the same despite the ten-week fasting period in the winter fasted group and autumnal fattening in the autumn fed group. The adipose tissue histopathology showed no accumulation of macrophages or the formation of crown-like structures in the analyzed samples. The hypoxia theory implies that abundance of white adipose tissue causes hypoxia in the adipose tissue of obese human subjects with subsequent infiltration of macrophages and activation of mast cells [44,45]. The absence of inflammatory structures in the subcutaneous and abdominal white adipose tissue and the lack of differences in circulating cytokines/chemokines between the groups indicate that the prolonged fasting as well as autumnal fattening did not produce any inflammatory response seen for example in humans [46,47]. This is further supported by the fact that the signal for N-acetylated glycoproteins, which generally reflects a change in inflammatory status, did not change in the plasma NMR profile [48].

Investigating key central food intake and metabolism regulating factors and the metabolic profiles, we found that the metabolite profile in plasma was different between the winter fed, winter fasted and autumn fed raccoon dog groups. The main variation between the groups was related to unsaturated lipoproteins and mobile saturated lipids which were more abundant in the fasted raccoon dog plasma samples compared to the fed groups, indicating that the animals were mobilizing and utilizing fat storages as energy source [49,50,51]. The fasted raccoon dogs exhibited also more glycerophosphocholines (diacylglycerophosphocholine) compared with the fed groups. The increased lactate and pyruvate concentration in the fed raccoon dogs compared with the fasted or less fed suggest the utilization of the Cori cycle/Warburg and Crabtree effect [52]. This is observed in metabolically challenging/stressful conditions such as obesity, cancer, inflammation, pregnancy and lactation, but also in relation to non-pathological growth. The increased lactate and pyruvate in the fed raccoon dogs suggests the continued maintenance of the adipose tissue storages. The various increased energy substrates including ketogenic body 3-hydroxybutyrate and ketogenic proteins albumin lysyl and creatinine, glycolytic pathway lactate, pyruvate, glucose, formate and the essential amino acid threonine and BCAAs (valine, isoleucine, leucine) are most likely due to the more active metabolism in the autumn fed raccoon dogs compared with the winter fasted raccoon dogs.

The untargeted UPLC-MS analysis revealed surprisingly few differentially expressed metabolites between the groups in single metabolite comparisons. From the 510 aligned positively ionized features, three metabolites were found with significantly different expressions. The metabolome database identifications suggest that two of these variedly expressed lipids belong to the larger lipid class of glycerophospholipids which includes e.g., glycerophosphocholines, glycerophosphoethanolamines and glycerophosphates. One of these glycerophospholipids (m/z 756.6) was significantly more expressed in the winter fasted compared with autumn fed and the database identification combined with the ^1^H NMR spectroscopy results suggest it as being glycerophosphocholine (diacylglycerophosphocholine). The second differentially expressed glycerophospholipids (m/z 858.6) was more abundant in the winter fed compared with the winter fasted. A feature (m/z 815.7) identified as sphingolipid was significantly more expressed in the winter fasted compared with autumn fed. Both glycerophospholipids and sphingolipids are main components in plasma membranes and other plasma lipoproteins [53]. Out of 78 negatively ionized features, penta amino acid (m/z 541.3) was significantly different between the groups, as it was increased in the two winter groups compared with the autumn fed, which may indicate a seasonal reduction in energy intake and increased protein catabolism in the winter groups compared with the autumn fed group.

In summary, the raccoon dog has developed regulatory mechanisms for autumnal fattening and prolonged fasting periods, enabling the species’ unique qualities in coping with the extreme environmental challenges. Further investigation of the molecular mechanisms might lead to novel molecular targets for food intake.

## 4. Materials and Methods

### 4.1. Animals

**Figure 9:**
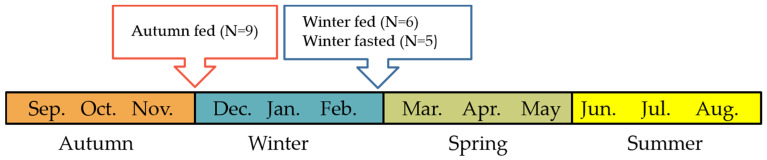
Timeline of the study setting and groups. The autumn fed group raccoon dogs were reared through the autumn hyperphagic phase and the experiment was finished before winter. The winter fasted raccoon dogs were fasted for ten weeks during winter while the winter fed raccoon dogs had ad libitum access to food. The experiment was finished for both of the winter groups after the fasting.

Three groups of farm-bred female raccoon dogs were reared at the Kannus Research Farm Luova Ltd. in northern Finland (elevation: 43.01 m, latitude: 63˚54′30″ N, and longitude: 23˚56′26″E) and were euthanized according to the Finnish legislation for the fur harvest at the end of the experiment. Blood and tissues samples were collected within 3 min after euthanasia. All animals used in the study were housed in roofed cages (150 × 107 × 70 cm) under natural temperature and photoperiod and had ad libitum access to water. In the study, we had three groups: winter fed, winter fasted and autumn fed. The winter fed group raccoon dogs (*n* = 6, female, age 8 months) had ad libitum access to commercial animal diet throughout the test period. Winter fasted group raccoon dogs (*n* = 5, age 8 months, female) were subjected to a ten-week fasting period during winter time between 22nd of December (5 h light, 18 h dark, 1 h dim light) and 1st of March (10 h light, 13 h dark, 1 h dim light). Autumn fed group raccoon dogs (*n* = 9, age > 10 months, female) also had ad libitum access to commercial animal diet and the animals were sampled on 1st of December (5 h light, 18 h dark, 1 h dim light) after the autumnal fattening period (Figure 9). The animals were fed a diet with high-energy content (5120 kcal/kg dry matter) to optimize body mass and fur size. The body weights of the winter fasted and winter fed raccoon dogs were recorded in the beginning (7th of December), three times during and at the end of the experiment (1st and 2nd of March). The autumn fed raccoon dogs were weighted only at the end of the experiment (1st of December) to avoid disturbing the hyperphagic phase. Both fed groups of raccoon dogs were fasted overnight before the blood and tissue sampling. EDTA blood was collected by cardiac puncture and plasma separated by centrifugation at 3000× *g* in +4 °C for 15 min and stored at −70 °C for further analyses. Tissue samples were excised and snap frozen with liquid nitrogen and stored at −70 °C prior to further analyses.

The experiment was approved by the National Committee for Animal Experimentation (licence no. ESLH-2008-06316/Ym-23) according to Finnish animal experimentation legislation (Act 62/2006).

### 4.2. Hypothalamic Gene Expression Determination

The expressions of NPY, OX2R, POMC and ObRb were determined in raccoon dog hypothalamus samples by quantitative real-time PCR. Total RNA was extracted from the frozen and homogenized hypothalamus samples with RNeasy Lipid Tissue Mini Kit (Qiagen, Hilden, Germany) according to the manufacturer’s protocol. iScript^TM^ cDNA Synthesis Kit (Bio-Rad Laboratories, Inc., Hercules, California, USA) was used to synthesize cDNA from 1 µg of RNA. The SYBR Green real-time PCR reactions were performed with ABI-PRISM 7300 sequence detection system (Applied Biosystems, ThermoFisher Scientific, Waltham, Massachusetts, USA) in a total volume of 30 µL. All samples were run as duplicates for each gene along with the internal control gene glyceraldehyde-3-phosphate dehydrogenase (GAPDH) in a 96-well plate. Sterile water was used as a negative control. Results were analyzed and calculated according to the manufacturer instructions. Since the raccoon dog genome has not been published to date, primers and antibodies were designed and tested on the basis of the publicly available dog genome (*Canis lupus familiaris*) [54]. Primers were designed with Primer Basic Local Alignment Search Tool (PRIMER BLAST). The dog sequences were acquired from the National Center for Biotechnology Information (NCBI) GenBank and the accession numbers for the sequences and primer sequences are listed in a supplementary table (Table S1: Primer sequences of NPY, POMC, ObRb, OX2R, GAPDH and the product sizes).

### 4.3. Metabolic Profiling

Raccoon dog plasma glucose-dependent insulinotropic peptide (GIP), GLP-1 and peptide YY (PYY) levels were determined using the Milliplex MAP Canine Gut Hormone Magnetic Bead Panel Assay (CAT# CGTMAG-98K, Millipore Corporation, Billerica, MA, USA) (intra-assay variation ≤ 5% CV, inter-assay variation ≤ 13% CV). Plasma granulocyte-macrophage colony-stimulating factor (GM-CSF), interferon gamma-induced protein, (IP-10), IL-4, IL-6, interleukin 8 (IL-8), interleukin 18 (IL-18), monocyte chemo-attractant protein-1 (MCP-1) and TNFα were analyzed with the Milliplex MAP Canine Cytokine/Chemokine Assay (CAT# CCYTO-90K, Millipore Corporation, Billerica, MA, USA)(intra-assay variation ≤ 15.6% CV, inter-assay variation ≤ 20,0% CV). Plasma resistin levels were determined by the Milliplex MAP Canine Adipokine Assay (CAT# CADPK-91K, Millipore Corporation, Billerica, MA, USA) (intra-assay variation ≤ 9.2% CV, inter-assay variation ≤ 16.6% CV). Plasma levels of pituitary derived peptides adrenocorticotropic hormone (ACTH), growth hormone (GH), BDNF, TSH, follicle-stimulating hormone (FSH) and luteinizing hormone (LH) were measured from plasma with Milliplex MAP Canine Pituitary Magnetic Bead Panel Assay (CAT# CPTMAG-96K, Millipore Corporation, Billerica, MA, USA) (intra-assay variation ≤ 3.26% CV, inter-assay variation ≤ 13.42% CV). Plasma samples were centrifuged at 10,000× *g* in +4 ˚C temperature for 10 min before analysis. All assays were performed utilizing a Bio-Plex 200 instrument based on Luminex xMAP technology (Bio-Rad Laboratories Inc., California, USA) [55]. The results were calculated with Bio-Plex Manager Software 6.0 with a five parameter logistical equation.

### 4.4. Metabolomics

The metabolic profile of the raccoon dog was assessed by utilizing two different metabolomics techniques: Proton nuclear magnetic resonance (^1^H NMR) spectrometry and ultra-performance liquid chromatography mass spectrometry (UPLC-MS). The protocol for the ^1^H NMR analysis of the plasma small metabolites is described in the supplementary (S1). Raccoon dog plasma samples were analyzed with ^1^H NMR and a multivariate analysis was conducted to a set of raccoon dog plasma samples representing different conditions. The three groups of winter fasted, winter fed and autumn fed indicated a separate clustering and were chosen for further analysis and reported on because they contain information about the conditions of interest (seasonal fattening and fasting). The corresponding PCA is presented in Figure S5 Loadings obtained by an OPLS-DA multivariate analysis on the NMR spectra comparing the three groups pairwise include metabolite identifications according to specific chemical shift and spectral peak profiles (Figure 4, Figure 5 and Figure 6) [56,57,58,59,60]. The height of the peak represents the abundance of the metabolite and the warmer coloring scheme (yellow, orange, red) indicate higher correlation value up to 1 within the group. The metabolite assignments were added in the figures for visualization.

Plasma lipid profiles were analyzed with untargeted UPLC-MS according to methodology described in the supplementary attachment (Supplement 1). The multivariate analysis of the UPLC-MS positive ionization mode results includes raccoon dog plasma samples from different conditions and the three groups of interest (winter fasted, winter fed and autumn fed) were analyzed further (Figure S6). Of the three groups, winter fasted in particular indicate a separate clustering in the PCA. The obtained data were processed using XCMS Online, a web-based platform for analyzing complex and extensive untargeted metabolomics data [61,62]. The datasets of the winter fasted, winter fed and autumn fed group raccoon dogs contained in total 510 aligned ion features, e.g., ions of certain m/z and rt, in positive mode and 78 ion features in the negative mode. The aligned ion features were compared individually between the groups by parametric multigroup comparisons (ANOVA, post hoc comparisons) with the XCMS Online platform. Cloud plots, total ion chromatograms and principal component analysis were generated with the XCMS online software were used to visualize the significantly differing features between groups and their expression intensities.

### 4.5. Histopathology of Adipose Tissue Vascularization and Inflammatory Structures

The histological structure on the adipose tissue was determined from 5 μm sections sliced from subcutaneous and abdominal adipose tissue samples with a cryostat microtome at −20 °C. The sections were stained with hematoxylin and eosin. The reagents used for tissue staining were Dako Hematoxylin (Agilent Technologies, Santa Clara, California, USA), Dako Eosin (Agilent technologies, Santa Clara, CA, USA) and Dako Bluing Buffer (Agilent Technologies, Santa Clara, CA, USA). The amount of nuclei was calculated from a 0.5 mm^2^ area and was used for an estimation of the amount and thus also the size and organization of the adipocytes. The shape of the adipocytes and inflammatory crown-like structures were determined visually by microscope. Vascularization of the adipose tissues was determined by counting the number of cross-sections or blood vessels from a 1 mm^2^ area. All the histological analyses were performed blinded by one examiner.

### 4.6. Statistical Analyses

Statistical analyses for the multiple comparisons was conducted with the one-way analysis of variance (ANOVA) followed by Tukey’s post hoc test using the IBM SPSS Statistics 21 Data Editor software (IBM, Armonk, NY, USA) for the hypothalamic peptide expression and plasma metabolite analyses. In the result analyses a *p*-value less than 0.05 was considered statistically significant. The results of the hypothalamic relative peptide expressions, plasma metabolites and adipose tissue histopathology results are presented as mean ± SEM. Statistically significant differences are indicated with * *p* < 0.05, ** *p* ≤ 0.01 and *** *p* ≤ 0.001. The statistical analyses used in the ^1^H NMR and UPLC-MS analyses are depicted in the more detailed methods in the supplementary attachment.

## 5. Conclusions

In raccoon dogs, orexigenic and anorexigenic signals were differentially expressed, allowing seasonal variation in the bodily fat stores. Plasma metabolic profiles remained remarkably stable between the seasons, despite the long fasting period. The voluntary reduction in the food intake in the winter suggest a lower set-point for body weight in the winter season which the animals try to maintain despite the access to food. Understanding the underlying mechanisms of how the lower body weight set-point is obtained and maintained despite access to food and the strong evolutionary drive to defend the body weight could reveal novel molecular targets for body weight control. Furthermore, investigation of the adipose tissue expandability without inflammation might reveal new mechanisms to counteract subacute inflammation in cases such as for human adiposity.

## Figures and Tables

**Table 1:** Body weight of the raccoon dogs at the beginning and end of the experiment. Statistically significant differences are indicated with ** *p* ≤ 0.01 and *** *p* ≤ 0.001. Beginning weights of the autumn fed raccoon dogs were not available (NA).

Weight (kg)	Winter Fasted (*n* = 5)	Winter Fed (*n* = 6)	Autumn Fed (*n* = 9)
Beginning of experiment	13.3 ± 1.1	14.0 ± 1.2	NA
End of experiment	9.0 ± 0.9 **	11.2 ± 0.9	12.3 ± 1.2 ***

**Table 2:** Measured plasma metabolites. Symbol # indicates values under assay detection limit and could not be measured.

		Winter Fasted (*n* = 5)	Winter Fed (*n* = 6)	Autumn Fed (*n* = 9)
Gut Hormones	GIP (pg/mL) PYY (pg/mL)	4.02 ± 1.02	7.16 ± 1.22	12.63 ± 4.13
		48.85 ± 4.07	65.41 ± 16.36	43.24 ± 6.70
Cytokines/Chemokines	GM-CSF (pg/mL) IP-10 (pg/mL) IL-8 (ng/mL) IL-18 (pg/mL) MCP-1 (pg/mL)	169.13 ± 40.04	102.48 ± 12.23	173.34 ± 72.44
		#	#	37.27 ± 16.77
		27.95 ± 7.11	16.96 ± 4.55	15.61 ± 2.12
		100.19 ± 29.39	54.89 ± 9.38	365.02 ± 108.50
		388.50 ± 72.11	187.37 ± 32.92	302.22 ± 95.58
Adipokines	Resistin (ng/mL)	56.75 ± 8.72	38.77 ± 3.98	42.48 ± 6.21
Pituitary Gland Peptides	ACTH (pg/mL) GH (ng/mL) FSH (ng/mL) LH (ng/mL)	209.86 ± 34.34	159.68 ± 43.70	159.50 ± 32.29
		0.46 ± 0.28	0.37 ± 0.16	0.14 ± 0.07
		91.90 ± 31.71	41.59 ± 17.96	76.66 ± 18.84
		1.03 ± 0.25	0.52 ± 0.15	1.12 ± 0.18

## Data Availability

Not applicable.

## Supplementary Materials

The following are available online at https://www.mdpi.com/article/10.3390/ijms22094965/s1, Supplementary methods information: Figure S1: Box- and whisker-plot 1, Figure S2: Box- and whisker-plot 2, Figure S3: Box- and whisker-plot 3, Figure S4: Box- and whisker-plot 4, Figure S5: PCA of the raccoon dog plasma NMR, Figure S6: PCA of the raccoon dog plasma UPLC-MS in positive ionization mode, Table S1: Primer sequences of NPY, POMC, ObRb, OX2R, GAPDH and the product sizes, Excel S1: Metabolite database annotations.
