# Supplementary material for: Seasonal Regulation of Metabolism: The Effect of Wintertime Fasting and Autumnal Fattening on Key Central Regulators of Metabolism and the Metabolic Profile of the Raccoon Dog (Nyctereutes Procyonoides)
Source: Int J Mol Sci. 2021 May 7;22(9):4965. doi: 10.3390/ijms22094965 (PMC8125260; doi:10.3390/ijms22094965)
Supplement: Supplementary file 1 [file ijms-22-04965-s001.zip › Proofreading - Supplements for zipping/Supplement_1_Proofread.pdf]

Supplement

^{1}H NMR spectroscopy methods

Sample preparation: Sample preparation and acquisition methods were annotated from previously published methods [1]. Aliquots of raccoon dog plasma (200 μL) were mixed with 400 μL of saline solution (0.9 % NaCl). The samples were allowed to stand for 10 min prior to centrifugation at 13000 rpm for 10 min in order to remove insoluble material. Aliquots of the supernatants (550 μL) from each plasma sample pipetted into 5 mm NMR tubes for ^{1}H NMR analysis.

Acquisition of ^{1}H NMR spectra: ^{1}H NMR spectra were acquired with a Bruker Avance 600 MHz spectrometer operating at 600.13 MHz for ^{1}H at 300 K. It was equipped with a 5 mm broad-band inverse configuration probe. Samples were randomly analysed in automation with a B-ACS 60 sample changer system. All plasma samples were analysed using water suppressed 1D NMR spectrum using the NOESYPRESAT pulse sequence (256 transients) and also a standard Carr-Purcell-Meiboom-Gill (CPMG) pulse sequence (128 transients). Irradiation of the solvent (water) resonance was applied during presaturation delay (2.0 s) for all spectra and also during the mixing time (0.1 s). The pulse sequence parameters including the 90° pulse (~ 10 μs), pulse frequency (~ 4.8 ppm), receiver gain (~ 200), and pulse powers were optimised for the first sample and used throughout the samples sets acquisition. The spectral width was 20 ppm for all spectra. The NMR was processed with an exponential line broadening of 1.0 Hz prior to Fourier transformation, which were collected with approximately 32 k real data points.

NMR spectral data processing: Data [-1.0 to 10.0 ppm] were imported into MATLAB 7.0 software (MathWorks, Natick, MA), where they were automatically phased, baseline corrected and referenced to the glucose found in the samples using scripts written in house. To reduce analytical variation between samples the residual water signal (4.67 – 4.98 ppm) was truncated from the data set. Probabilistic quotient normalisation was used as it is considered a robust method to account for the dilution of complex biofluids [2], after the residual water peak was truncated. Assignment of endogenous plasma water metabolites was made by reference to published literature data [3,4].

Statistical methods and software: Following the processing of the NMR data multivariate statistical analysis was performed using both SIMCA-P 12.0 and MATLAB 7.0. Principal component analysis (PCA) [5] which aided the identification of unusual spectra and obvious trends in the metabolite profiles was performed using SIMCA-P 12.0. PCA analysis was conducted to a set of raccoon dog plasma samples representing different conditions and the three groups of (winter fasted, winter fed and autumn fed) were chosen for further analysis (Figure S1). Orthogonal partial-least squares discriminant analysis (OPLS-DA) using both univariate and mean centered scaling was used to identify specific metabolites pertaining to a particular sample group. All OPLS models were run through permutation testing to assess the validity of the supervised model.

UPLC-MS methods

Sample preparation: Raccoon dog plasma samples (25 μL) were each extracted with 100 μL of chloroform/methanol mixture (2:1), which were then allowed to stand for 5 min at room temperature and vortexed for 30.0 s. After centrifugation at 12000 rpm for 5 min at 4 °C the lower organic phase was collected and allowed to evaporate under vacuum conditions until dry. Immediately before the analysis the extracted plasma lipid samples were diluted with 250 μL isopropanol/acetonitrile/water mixture (2:1:1).

Liquid chromatography (LC) conditions: Samples were analyzed by UPLC–MS with Waters Acquity UPLC (Waters, Milford, MA). The LC was conducted with ACQUITY UPLC system with ACQUITY UPLC CSH C_{18} column (2.1 x 100 mm, 1.7 μm; 55 °C; 400 μL/min flow rate). Mobile phase A reagents include acetonitrile/water (60:40) with 10 mM ammonium formate and 0.1 % formic acid and mobile phase B include isopropanol/acetonitrile (90:10) with 10 mM ammonium formate and 0.1 % formic acid. The system injection volume is 5 μL.

Mass spectrometry (MS) conditions: Mass spectrometer SYNAPT G2 HDMS with positive and negative electrospray ionization (ESI) mode. Capillary voltage was 2.0 KV for positive and 1.0 KV for negative ionization mode. Other conditions include cone voltage 30 V, desolvation temperature 550 °C and gas (900 L/h), source temperature 120 °C. Acquisition range varied between 100 and 2000 m/z.

UPLC-MS data processing: Multivariate analysis of the raccoon dog UPLC-MS positive ionization mode results was performed using SIMCA 13.0 and the PCA is presented in figure S2. The PCA includes raccoon dog plasma samples from different conditions and the three groups of interest (winter fasted, winter fed and autumn fed) were analyzed further. Pooled quality controls were included in the analysis. The acquired UPLC-MS data was processed using XCMS Online, a web-based platform for analyzing complex and extensive untargeted metabolomics data [6]

Supplementary figures

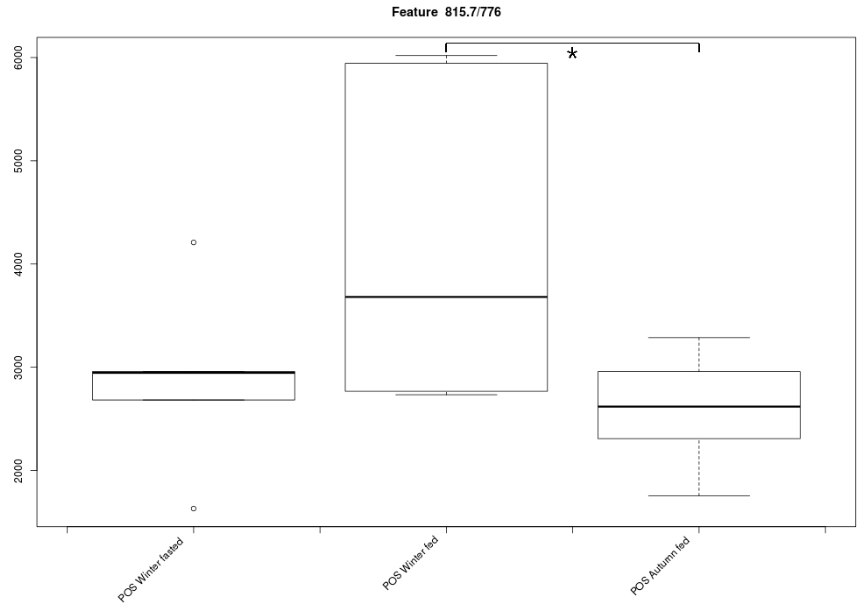

Figure S1: Box- and whisker-plot 1. Box- and whisker-plot of a differentially expressed POS feature m/z 815.7 and RT 12.94. Box-and-Whisker plot representing the area under the curve of intensity of the feature with meridian, intensity and deviations as well as outlier values presented in the picture. Statistically significant differences indicated with *p < 0.05.

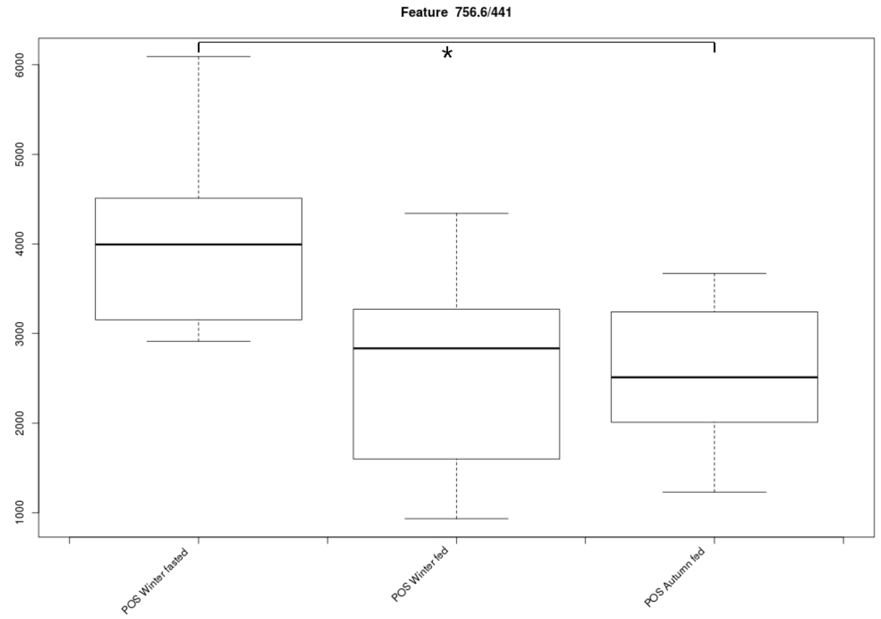

Figure S2: Box- and whisker-plot 2. Box-and-Whisker plot of a differentially expressed POS feature m/z 756.6 and RT 7.35. Box-and-Whisker plot representing the area under the curve of intensity of the feature with meridian, intensity and deviations as well as outlier values presented in the picture. Statistically significant differences indicated with *p < 0.05.

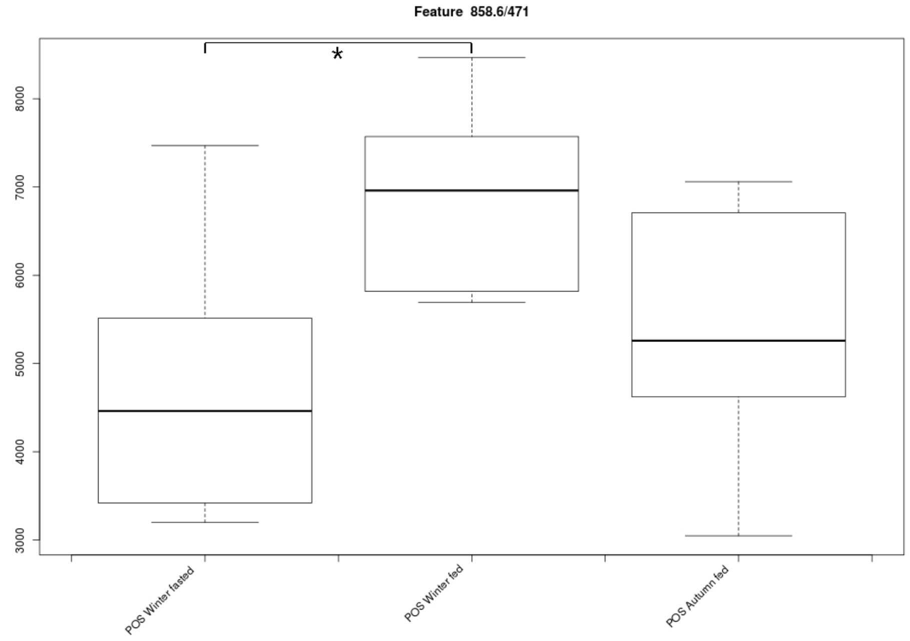

Figure S3: Box- and whisker-plot 3. Box-and-Whisker plot of a differentially expressed POS feature m/z 858.6 and RT 7.85. Box-and-Whisker plot representing the area under the curve of intensity of the feature with meridian, intensity and deviations as well as outlier values presented in the picture. Statistically significant differences indicated with *p < 0.05.

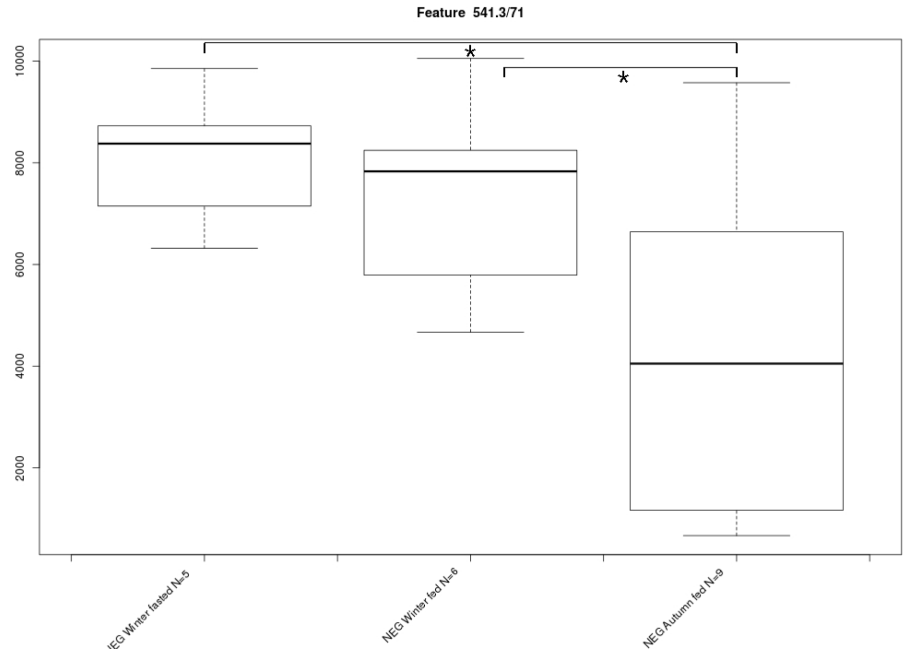

Figure S4: Box- and whisker-plot 4. Box-and-Whisker plot of a differentially expressed NEG feature m/z 541.3 and RT 1.19. Box-and-Whisker plot representing the area under the curve of intensity of the feature with meridian, intensity and deviations as well as outlier values presented in the picture. Statistically significant differences indicated with *p < 0.05.

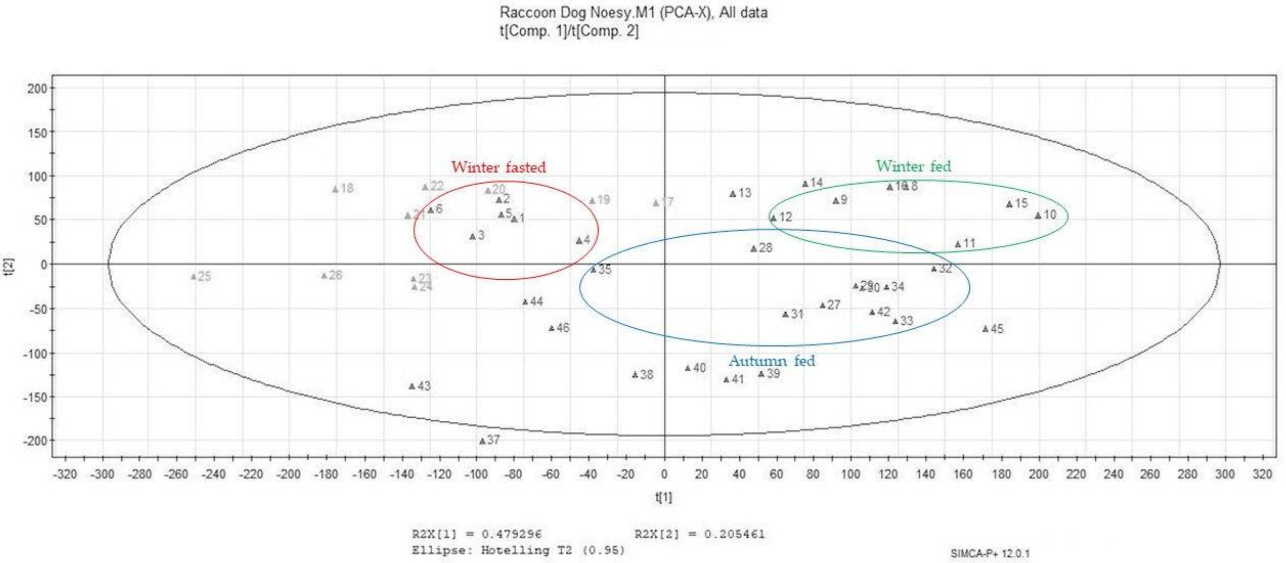

Figure S5: PCA of the raccoon dog plasma NMR. Winter fasted (1,2, 4-5), winter fed (8-12) and autumn fed (27-36).

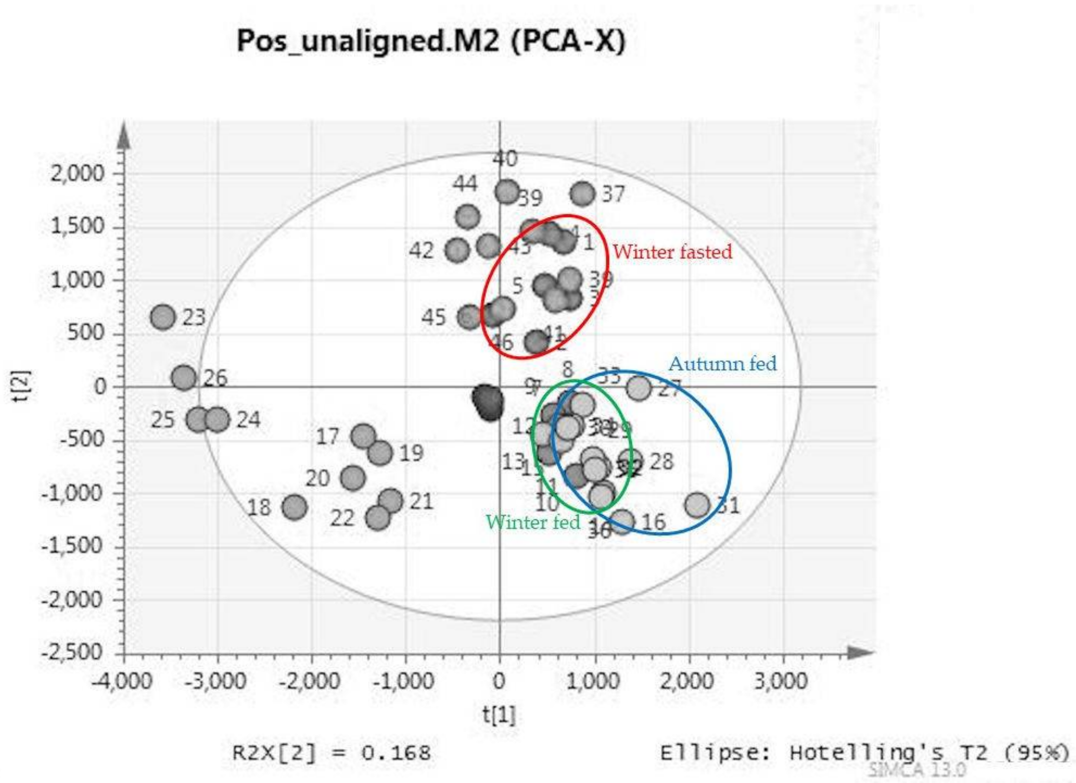

Figure S6: PCA of the raccoon dog plasma UPLC-MS in positive ionization mode. Winter fasted (1,2, 4-5), winter fed (8-12) and autumn fed (27-36). Pooled quality controls are clustered in the middle.

Supplementary table

Table S1: Primer sequences of NPY, POMC, ObRb, OX2R, GAPDH and the product sizes.

**Gene**	**GenBank Accession No.**	**Forward 5′ → 3′**	**Reverse 5′ → 3′**	**Product length (bp)**
NPY	XM_532492*	TCACCAGGCAGAGGTATGGA	CCAGCCTAGTTCTGGGAACG	99
POMC	XM_844370*	CCTCTGTGGAAGTGAGTGGC	GATGCACGCCAGCAGGTTAC	128
ObRb	AY753649#[a]	TGAGGAAGAGCAAGGGCTTA	AATGGAAGGTGTGGCAAAAT	165
OX2R	NM_001002933	AGTGTGTGATGAACGCTGGGGT	GATGTTCCAGGGATCTGGCGAC	153
GAPDH	NM_001003142	GCCTGGAGAAAGCTGCCAAA	GTCGAAGGTGGAAGAGTGGG	146

*Predicted, #Partial cds, [a]Primers used in publication [7].

References

1. Beckonert, O.; Keun, H.C.; Ebbels, T.M.; Bundy, J.; Holmes, E.; Lindon, J.C.; Nicholson, J.K. Metabolic profiling, metabolomic and metabonomic procedures for NMR spectroscopy of urine, plasma, serum and tissue extracts. *Nat Protoc* **2007**, *2*, 2692-2703, doi:10.1038/nprot.2007.376.

2. Dieterle, F.; Ross, A.; Schlotterbeck, G.; Senn, H. Probabilistic quotient normalization as robust method to account for dilution of complex biological mixtures. Application in1H NMR metabonomics. *Anal Chem* **2006**, *78*, 4281-4290, doi:10.1021/ac051632c.

3. Nicholson, J.K.; Foxall, P.J.D.; Spraul, M.; Farrant, R.D.; Lindon, J.C. 750 MHz 1 H and 1 H 13 C NMR spectroscopy of human blood plasma. *Anal Chem* **1995**, *67*, 793-811, doi:10.1021/ac00101a004.

4. Tang, H.; Wang, Y.; Nicholson, J.K.; Lindon, J.C. Use of relaxation-edited one-dimensional and two dimensional nuclear magnetic resonance spectroscopy to improve detection of small metabolites in blood plasma. *Anal Biochem* **2004**, *325*, 260-272, doi:10.1016/j.ab.2003.10.033.

5. Lindon, J.C.; Holmes, E.; Nicholson, J.K. Pattern recognition methods and applications in biomedical magnetic resonance. *Prog Nucl Magn Reson Spectrosc* **2001**, *39*, 1-40, doi:10.1016/S0079-6565(00)00036-4.

6. Gowda, H.; Ivanisevic, J.; Johnson, C.H.; Kurczy, M.E.; Benton, H.P.; Rinehart, D.; Nguyen, T.; Ray, J.; Kuehl, J.; Arevalo, B.; Westenskow, P.D.; Wang, J.; Arkin, A.P.; Deutschbauer, A.M.; Patti, G.J.; Siuzdak, G. Interactive XCMS online: Simplifying advanced metabolomic data processing and subsequent statistical analyses. *Anal Chem* **2014**, *86*, 6931-6939, doi:10.1021/ac500734c.

7. Knudson, J.D.; Dincer, ÜD.; Zhang, C.; Swafford Jr., A.N.; Koshida, R.; Picchi, A.; Focardi, M.; Dick, G.M.; Tune, J.D. Leptin receptors are expressed in coronary arteries, and hyperleptinemia causes significant coronary endothelial dysfunction. *Am J Physiol Heart Circ Physiol* **2005**, *289*, doi:10.1152/ajpheart.01159.2004.
